# Trends in Adolescent Treatment Admissions for Marijuana in the United States, 2008–2017

**DOI:** 10.5888/pcd17.200156

**Published:** 2020-11-19

**Authors:** Jeremy Mennis

**Affiliations:** 1 Department of Geography and Urban Studies, Temple University, Philadelphia, Pennsylvania

**Figure:**
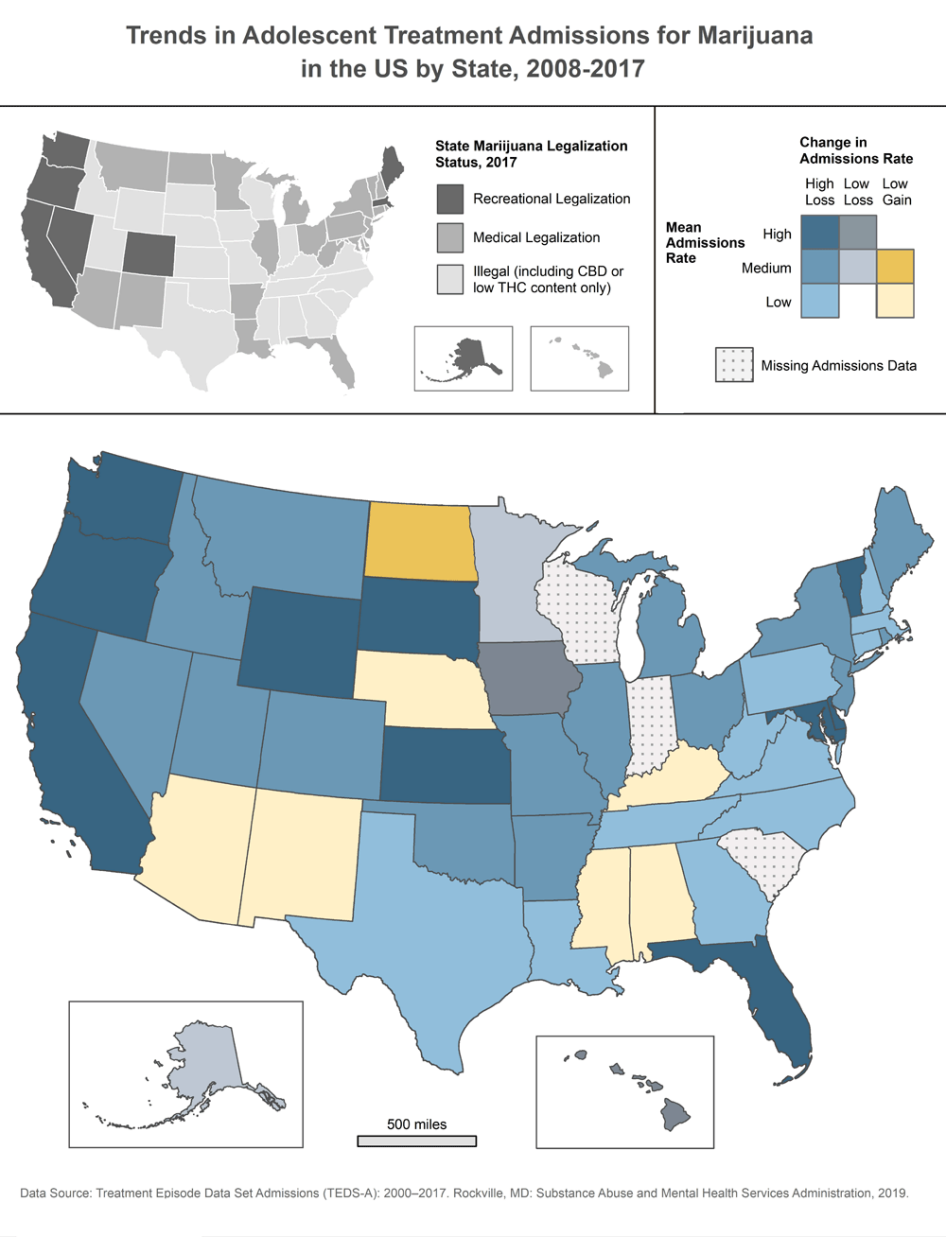
Map. The main map shows the 2008–2017 mean admissions rate for each state. Declines in mean admissions rates over time are shown in blue and gray, and increases are shown in orange. States with higher admissions rates are darker and states with lower rates are lighter. Marijuana legalization status appears in the inset map. Abbreviations: CBD, cannabidiol; THC, tetrahydrocannabinol.

## Background

The legalization of marijuana for medical and recreational uses is expanding in the United States and internationally. Although legalization may address some of the social ills associated with the history of marijuana criminalization (1), the movement toward marijuana legalization has substantial implications for public health, especially for youth. Even with age restrictions for use, legalization may increase the availability and social acceptability of marijuana among youth (2). Adolescent marijuana use, particularly heavy use, is associated with a host of negative health outcomes, including mental health problems and cannabis use disorder (CUD) (3). Although US adolescent marijuana use has not recently increased, the perception of marijuana use as harmful has substantially declined (4). In addition, although recent research suggests that recreational legalization has not prompted a rise in CUD treatment among youth (5), incidence of CUD may have risen (6). Monitoring trends in youth marijuana use, CUD, and treatment is necessary to guide public health responses to rapidly evolving marijuana laws. This research contributes to such monitoring by mapping state-level changes in admissions for substance use disorder treatment for marijuana use among US adolescents from 2008–2017, which spans the beginning of recreational legalization in the United States in 2012.

## Data and Methods

Annual 2008–2017 data on substance use disorder treatment admissions among adolescents (aged 12–17) were extracted from the Treatment Episode Data Set: Admissions (TEDS-A) (7), a national compilation of admissions to publicly funded substance abuse treatment facilities. Consistent with prior research (8), only observations with no prior admissions were retained to ensure that each admission represents a single individual. Wisconsin, Indiana, and South Carolina were excluded because of a large amount of missing data (including missing prior admission information). Seven other states with 3 or fewer years of missing data each were included in the analysis. We calculated the number of annual treatment admissions for each state where the primary substance used was marijuana (or hashish or other cannabis preparation), divided by the total number of adolescents (derived from US Census Bureau annual American Community Survey data files) to yield the annual treatment admissions rate (per 10,000 adolescents). For each state, the slope of the annual change in admissions rate (ie, the linear rate of increase or decline over the study period) was calculated, using within-state standardized values to facilitate comparison among states. Thus, a slope of 0.10 indicates an annual admissions rate increase of 10% of 1 standard deviation for that state. We also calculated the mean admissions rate over the study period for each state to facilitate comparison of the magnitude of rates among states.

The map depicts both the slope of the admissions rate (ie, admissions rate gain or loss) and the mean of the admissions rate (ie, admissions rate magnitude) for each state. We used a sequential-diverging, bivariate color scheme in a choropleth map (9), where sequential variation in darkness is used to extend a univariate color scheme to a bivariate context (10). Here, blue is used to indicate a decrease in admissions rate, and orange is used to indicate an increase. A modified 3-class, equal interval classification was used, where the range in slope (min = –0.42, max = 0.19) is classified using breaks at −0.20 and 0.00, with greater saturation indicating a greater departure from zero slope. Color choices were derived from ColorBrewer 2.0 (11). The state mean admissions rate is expressed by altering the darkness of the hue, where states with higher admissions rates appear darker. A 3-class, standard deviation classification was used, where the range in the admissions rate mean (min = 0, max = 233) is classified using breaks at 30 and 72, such that the middle class extends 1 standard deviation centered on the mean. Marijuana legalization status in 2017 is shown using an inset map. Data manipulation was conducted in SPSS version 25 (IBM) and Excel 2016 (Microsoft Corporation). The map was created using ArcGIS Desktop 10.6.1 (Esri).

## Highlights

The map, visually dominated by blue tones, clearly shows that adolescent treatment admissions for marijuana declined in most of states. The mean annual admissions rate for all states declined over the study period by nearly half, from 60 (admissions per 10,000 adolescents) in 2008 to 31 in 2017, with state admissions rate slopes ranging from −0.42 to 0.19 (median = –0.28). State admissions rates in 2008 ranged from fewer than 1 to 218 (median = 52); in 2017 they ranged from fewer than 1 to 167 (median = 21). Admissions rates increased over the study period in only 7 states, 6 of which (excepting North Dakota) have relatively low mean admissions rates (states colored lighter orange). Low mean admissions rates tend to occur in a loose band extending from the Southwest through the South, Appalachia, and into parts of New England. All 12 states in the high mean admissions rate class sustained admissions declines, with 10 of those states having declines in the steepest category (states colored darkest blue). Consistent with prior research on medical marijuana and adolescent marijuana use (12), medical legalization status does not appear to correspond to treatment admission trends. Notably, however, 7 of 8 states with recreational legalization during the study period fall into the class with the steepest level of admissions decline.

## Action

To our knowledge, this map is the first to illustrate state level trends in adolescent treatment admissions for marijuana, and the trends depicted can inform public health responses to changing marijuana laws. Possible causes for the overall decline, and variations among states, in admissions trends include changes in attitudes toward marijuana, as well as differences among states in marijuana use and incidence of CUD, as well as in socioeconomic status, treatment availability, and health insurance (5). Whatever the causes of the observed patterns, however, this research suggests that a precipitous national decline in adolescent treatment admissions, particularly in states legalizing recreational marijuana use, is occurring simultaneously with a period of increasing permissiveness, decreasing perception of harm, and increasing adult use, regarding marijuana (4,13). These trends indicate the need for sustained vigilance in the prevention and treatment of youth CUD during this period of expanding marijuana legalization.
